# Role of the Short Distance Order in Glass Reactivity

**DOI:** 10.3390/ma11030415

**Published:** 2018-03-11

**Authors:** María Vallet-Regi, Antonio J. Salinas

**Affiliations:** 1 Departamento de Química en Ciencias Farmacéuticas, Universidad Complutense de Madrid, Instituto de Investigación Sanitaria Hospital, 12 de Octubre imas12, 28040 Madrid, Spain; 2 Networking Research Center on Bioengineering, Biomaterials and Nanomedicine (CIBER-BBN), 28040 Madrid, Spain

**Keywords:** bioactive glasses, nanostructure, short distance order, HRTEM, NMR spectroscopy

## Abstract

In 2005, our group described for the first time the structural characterization at the atomic scale of bioactive glasses and the influence of the glasses’ nanostructure in their reactivity in simulated body fluids. In that study, two bioactive sol-gel glasses with composition 80%SiO_2_–20%CaO and 80%SiO_2_–17%CaO–3%P_2_O_5_ (in mol-%) were characterized by High-Resolution Transmission Electron Microscopy (HRTEM). Such characterization revealed unknown features of the glasses’ structure at the local scale that allowed the understanding of their different in vitro behaviors as a consequence of the presence or absence of P_2_O_5_. Since then, the nanostructure of numerous bioactive glasses, including melt-prepared, sol-gel derived, and mesoporous glasses, was investigated by HRTEM, Nuclear Magnetic Resonance (NMR) spectroscopy, Molecular Dynamics (MD) simulations, and other experimental techniques. These studies have shown that although glasses are amorphous solids, a certain type of short distance order, which greatly influences the in vitro and in vivo reactivity, is always present. This paper reviews the most significant advances in the understanding of bioactive glasses that took place in the last years as a result of the growing knowledge of the glasses’ nanostructure.

## 1. Introduction

Bioactive glasses (BGs) have been widely investigated in the last few decades for biomedical applications because they are biocompatible and bond to bone when implanted [1,2,3,4,5,6,7]. BGs can be classified as: Melt Prepared Glasses (MPGs), first reported in 1971 [8], Sol-Gel Glasses (S-GGs), in 1991 [9], and Mesoporous Bioactive Glasses (MBGs), in 2004 [10]. MPGs are dense materials, whereas S-GGs and MBGs are highly porous materials. Furthermore, MBGs exhibit surface area and volume of pores nearly double those of analogous S-GGs because they are synthesized in the presence of surfactants. Moreover, MBGs show mesopores arrangements in an extremely narrow pore size distribution.

In vitro assays in Simulated Body Fluid (SBF) [11,12,13] have played an important role in the design and evaluation of new BGs. The validity of these assays relies on the assumption that the apatite-forming ability of a biomaterial in SBF can be used as a predictor of a bioactive response when implanted. In 2014, Zadpoor reported that this assumption was valid in 25 of 33 studies of biomaterials reviewed [14]. In the majority of failure cases (i.e., 5/8), none of the biomaterials were able to form apatite in vitro, while they showed an in vivo bioactive behavior. However, for silica-based BGs, which this article addresses, the validity of this assumption is complete. Therefore, assays in SBF have also allowed the selection of the most promising BGs for evaluation in animal models. Moreover, these assays allowed the proposal of mechanisms of formation of the hydroxycarbonate apatite (HCA) layer, indicative of an in vitro bioactive response, as a consequence of variations in the glass composition or in the assay conditions.

**Figure 1:**
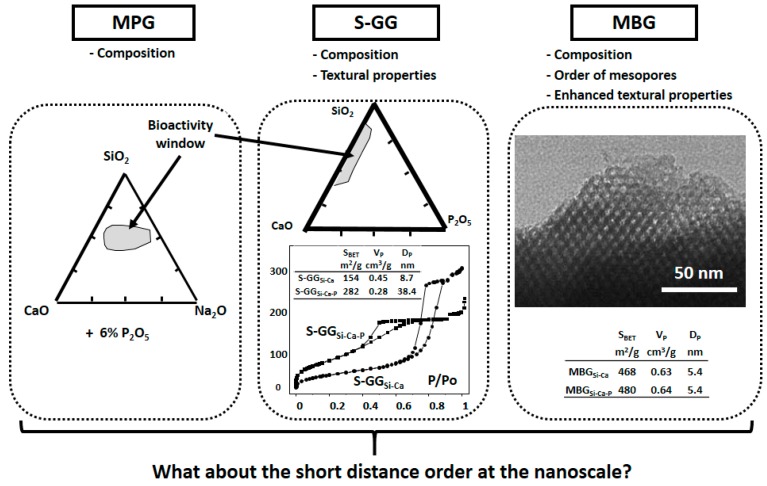
Properties which determine the bioactive response of the three families of BGs: MPGs, S-GGs, and MBGs. In the last decade, the importance of the nanostructure in this response for the three families of BGs has been also stressed.

Figure 1 shows the properties traditionally investigated as responsible for the bioactive response of each family of BGs. As is observed, for the dense MPGs, the in vitro bioactive response only depends on the glass composition. However, in S-GGs, the textural properties, i.e., surface area and porosity, must be also considered. Indeed, these properties produced a shift in the bioactivity window towards compositions richest in SiO_2_. Finally, the enhanced textural properties of MBGs accelerate their bioactive response.

Moreover, in vitro assays in SBF were the key to understanding the relationship between BG nanostructure and reactivity, first reported by our group in 2005 [15] and whose progress since then will be reviewed in this paper.

## 2. Traditional Characterization of Bioactive Glasses

Traditionally, BGs were characterized before and after being soaked in SBF by X-Ray diffraction (XRD), Fourier Transform Infrared (FTIR) spectroscopy, Scanning Electron Microscopy (SEM), Energy Dispersive X-Ray (EDX) spectroscopy, Solid State Nuclear Magnetic Resonance (NMR) spectroscopy, X-ray Fluorescence (XRF), Transmission Electron Microscopy (TEM), electron diffraction (ED), X-ray Photoelectron Spectroscopy (XPS), and nitrogen adsorption. After a certain period soaked in SBF, the HCA formation on the glass surface indicative of a bioactive behavior is established when: (i) XRD and ED patterns can be assigned to an apatite-like phase, (ii) the FTIR spectrum contains two bands characteristic of phosphate in a crystalline environment (at 603 and 567 cm^−1^) and of carbonate groups (at 1440 and 875 cm^−1^) [16], and (iii) SEM and TEM show the typical needle-like shape particles of apatite, and EDX spectra indicates the new material formed is mainly composed of calcium and phosphorus. That way, the shorter the time of formation of HCA, the quicker the in vitro bioactive response of an investigated glass.

**Figure 2:**
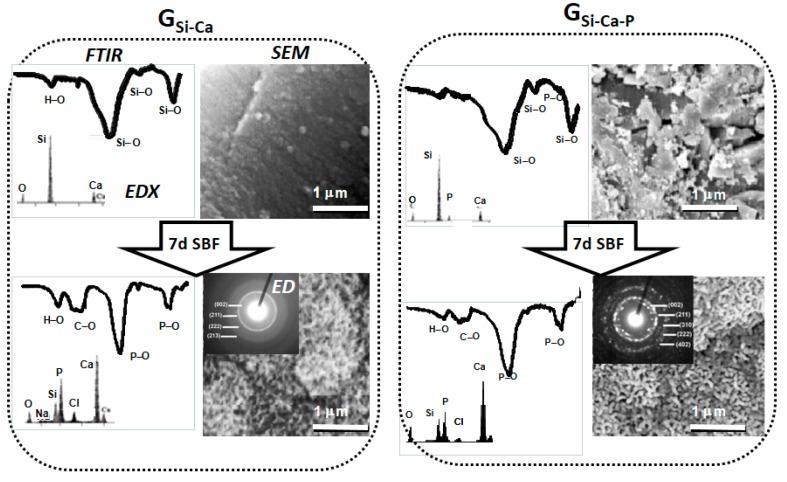
FTIR and EDX spectra, SEM images, and ED patterns of two bioactive glasses before and after being soaked for 7 days in SBF. **Left**: a P-free glass, G**_Si–Ca_**. **Right**: a glass with analogous composition but including 3 mol-% of P_2_O_5_, G**_Si–Ca–P_**.

Figure 2 shows the assessment of the in vitro bioactive response of two S-GGs, the first one with composition 80%SiO_2_–20%CaO (G**_Si–Ca_**), and the other one containing 3 mol-% of P_2_O_5_ at the expense of the CaO (G**_Si–Ca–P_**). SEM micrographs showed that after 7 days in SBF, the surfaces of both glasses were coated by a new material. The new bands observed by FTIR established that the new material contained PO_4_^3−^ groups in a crystalline environment, CO_3_^2−^ groups, and H_2_O. In addition, EDX analysis showed that the new material was mainly composed of calcium and phosphorous. Finally, the ED patterns were indexed to a poorly crystallized apatite phase with somewhat bigger crystal size in G**_Si–Ca–P_** deduced by the presence of dots together with the rings [17]. All these results yield the conclusion that both glasses exhibited in vitro bioactivity because they were coated by HCA. However, the SEM images and the ED patterns indicated a bigger size of the HCA crystals formed on G**_Si–Ca–P_**.

**Figure 3:**
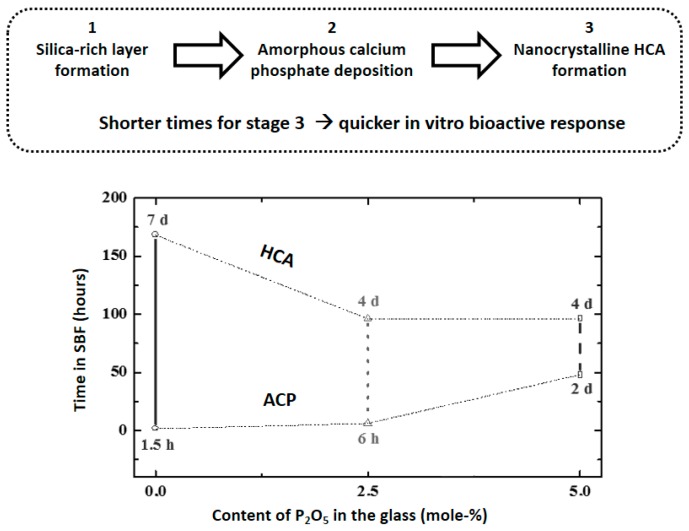
**Top**: Main outcomes after soaking a BG in SBF. **Bottom**: The times required for stages 2 and 3 for three silica-based sol-gel glasses containing, respectively, 0, 2.5 and 5.0 mol-% P_2_O_5_.

These results suggested that the inclusion of P_2_O_5_ in the glasses modified the HCA formation and pushed us to perform a study in three S-GGs containing 25 mol-% of CaO and 0, 2.5 and 5% of P_2_O_5_, respectively (rest SiO_2_) [18]. Figure 3, top, shows the previous events that yielded the HCA crystallization: formation of a silica-rich layer and deposition of amorphous calcium phosphate (ACP) [2]. Figure 3, bottom, shows the times required to detect the formation of ACP and HCA for each glass.

As is observed, the increase of P_2_O_5_ in the glasses decreases the required time for the HCA formation, that is, increases the bioactivity, although it retards the ACP formation. These results prompted us to propose different mechanisms for the HCA formation depending on whether or not the glass contained phosphorus. However, the traditional characterization methods did not allow the confirmation of these mechanisms. For this reason, we planned the nanostructural characterization of BGs with and without P as is described in the following section.

## 3. Nanostructure of Bioactive Glasses and Relationship with Their Bioactivity

### 3.1. Nanostructure of Bioactive Glasses from High-Resolution Transmission Electron Microscopy Analysis

**Figure 4:**
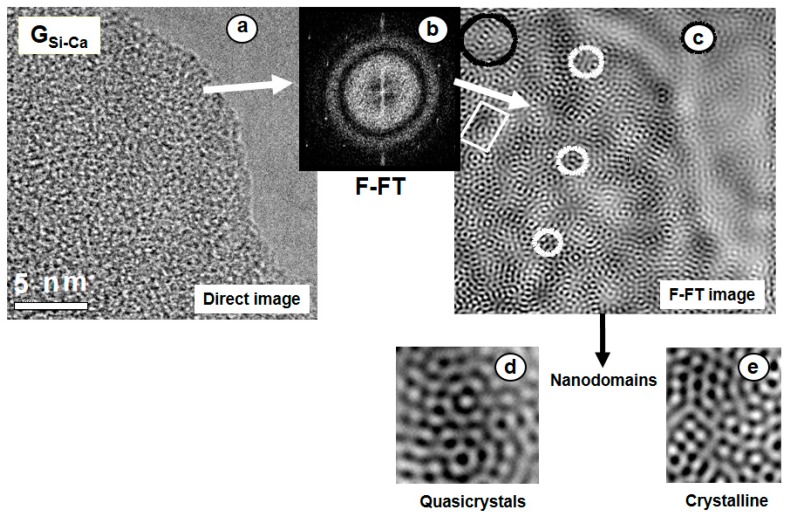
(**a**) Direct HRTEM image of G**_Si–Ca_** glass where no specific structural feature is revealed. (**b**) The Filtered Fourier-Transform (F-FT) of the digitized image. (**c**) Noise-free reconstructed image shows the presence of crystalline and quasi-crystalline nanodomains more clearly noticeable in the magnified images (**d**,**e**).

Two S-GG bioactive glasses, the first one P-free bioactive, G**_Si–Ca_**, and the second one including 3% P_2_O_5_, G**_Si–Ca–P_**, whose bioactivity was assessed in Figure 2, were characterized by High-Resolution Transmission Electron Microscopy (HRTEM) [15]. The high resolution image of G**_Si–Ca_** in Figure 4a shows the typical contrast of an amorphous material but does not reveal any specific structural feature. However, this image can be filtered following the process: (i) digitizing the image, (ii) obtaining the Fourier transform (FT), (iii) filtering the FT by placing small windows around the fundamental spots, Figure 4b, and (iv) performing the inverse FT. The obtained noise-free image, Figure 4c, allowed us, for the first time, to reveal the structure at the atomic scale of a bioactive S-GG. The observed dots were correlated with positions of the tetrahedral units [SiO_4_^4−^] and an average distance of 0.53 nm between the tetrahedra was determined for this P-free BG. The image shows local quasi-crystalline and crystalline blocks as well as some kind of chain connecting these different blocks. Figure 4d,e show the magnification of some regions of Figure 4c.

**Figure 5:**
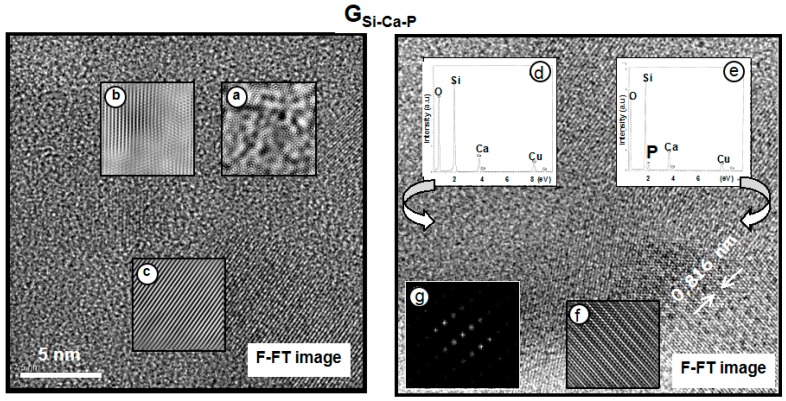
**Left**: Fourier-Filtered HRTEM of G**_Si–Ca–P_**. (**a**) The magnification of an amorphous region and of two crystalline areas (**b**,**c**). **Right**: (**d**) EDX spectra of an amorphous region, depleted in P, and of a crystalline region (**e**), rich in P. (**f**) Magnification of the right lower corner of a crystalline region and its corresponding FT pattern (**g**).

At Figure 5, left, the F-FT image of G**_Si–Ca–P_** shows a glassy matrix including some crystalline domains. The magnification of the amorphous part, Figure 5a, shows similar structural features as in G**_Si–Ca,_** but the average distance between [SiO_4_^4−^] tetrahedra is now 0.36 nm, that is, quite shorter than the 0.53 nm measured for the P-free glass. Moreover, Figure 5b,c show the magnified images of two crystalline areas with interplanar spacing close to 0.26 nm. Figure 5, right, superimposed with the image, shows now the EDX spectra of an amorphous region, Figure 5d, and of a crystalline region, Figure 5e. In addition, the magnified image of a crystalline area, Figure 5f, and its ED pattern, Figure 5g, are also included. EDX analyses revealed that phosphorus was only present in the crystalline regions and that amorphous areas were depleted in calcium. Particularly interesting is the ordered area at the bottom right, whose magnified image is depicted in Figure 5f, and its FT pattern in Figure 5g.

The FT diffraction pattern (Figure 5g) allowed the identification of the nanocrystalline phase. This pattern is characteristic of a material with rhombohedral symmetry like β-tricalcium phosphate (β-TCP). However, the decrease of symmetry was explained by the inclusion of small amounts of silicon in the unit cell, seen in Figure 5e [19]. Thus, Figure 5g was indexed on the basis of an R3m space group with unit cell parameters somewhat smaller than those of β-TCP, that is a = 0.7135 nm and c = 2.5586 nm.

Therefore, in spite of G**_Si–Ca–P_** exhibiting a glassy matrix containing some crystalline domains like G**_Si–Ca_**, in the P-containing glass, the amorphous regions were depleted in calcium and exhibited shorter distances between the [SiO_4_^4−^] tetrahedra. These facts were explained considering that in G**_Si–Ca–P_**, the Ca^2+^ ions were out of the glass network and bonded to phosphate in nanocrystals identified as Si-doped β-TCP with crystallites smaller than 10 nm. Thus, HRTEM showed phase separation of silicate-rich (amorphous) and phosphate-rich (nanocrystalline) regions in the P-containing glass. 

### 3.2. Mechanism of Bioactivity of Bioactive Glasses Explained from Their Nanostructure

The characterization of BG by HRTEM allowed the determination of the nanostructural factors relevant for the in vitro deposition of ACP and HCA. This characterization revealed that BGs exhibit short distance order features that greatly influence the glass dissolution and reactivity and, consequently, the formation of HCA in SBF. Moreover, HRTEM explained discrepancies in the kinetics of formation of HCA along the SBF tests (Figure 3). Calcium phosphate nanocystals in P-containing BG increased the silica network repolymerization, unfavorable for the initial glass reactivity. For this reason, the ACP formation was retarded in P-containing glasses (see Figure 3). However, these nanocrystals have the positive effect of behaving as nuclei, accelerating the HCA crystallization in the P-containing glasses. Therefore, the in vitro behavior of binary G**_Si–Ca_** was conditioned by the location of the Ca^2+^ ions in an amorphous silica network, whereas in G**_Si–Ca–P_** they were present in silicon-doped calcium phosphate nanocrystals. This fact decreased the Ca^2+^ released to the medium when increasing the P_2_O_5_ content, which retarded ACP formation.

HRTEM explained the results from traditional characterization methods, for instance, the two very low-intensity bands at 604 and 567 cm^−1^ in the FTIR spectrum and the diffuse reflections at 26° and 32° in 2θ in the XRD pattern of G**_Si–Ca–P_** before being soaked in SBF [18]. Both results suggested the presence of nanocrystalline phosphate nuclei in G**_Si–Ca–P_** that were visualized and identified by HRTEM. The N_2_ adsorption measurements also have shown that the additions of P_2_O_5_ produced the same effect of calcium decreasing the CaO content in a SiO_2_–CaO glass. Again, HRTEM characterization also explained this fact because it showed the great affinity of phosphorus for calcium to form nanocrystals, consequently retaining the Ca^2+^ ions from the glass network.

### 3.3. Recent Advances in the Characterization of the Nanostructure of Bioactive Glasses

The pathway opened in 2005 by our group was followed by other authors. Thus, in 2007, Tilocca and Cormack confirmed by Molecular Dynamics simulations the structural effects of phosphorus inclusion in bioactive silicate glasses obtained by HRTEM. Their study was focused on the structural features that can have a role in the bioactive mechanism of dissolution and bone bonding [20]. They concluded that the higher affinity of Na^+^ and Ca^2+^ cations for coordinating phosphate than of silicate and the P–O–Si linkages formation increased repolymerization of the silicate network with increasing P_2_O_5_. This negative effect of the P inclusion was counterbalanced by the concomitant increase for free orthophosphate groups, whose fast release is deemed to enhance the bioactivity. Thus, although the presence of P_2_O_5_ could reduce the glass bioactivity, the favorable balance between the effects mentioned should result in a positive effect of partial substitution of Si by P. Later on, Tilocca published a paper in 2009 reviewing advances in computer modelling of bioactive glasses based on MD simulations, which were starting to unveil key structural features of these materials [21].

Other groups, such as Aguiar et al. [22], investigated structural aspects of MPGs by HRTEM, FTIR, and ^29^Si and ^31^P MAS NMR in 2008. They concluded that P was present in nanocrystals with interplanar distances in the range 0.21–0.26 nm. These glasses developed a surface calcium phosphate-rich layer without detecting the presence of an intermediate silica-rich layer. They suggested that the phosphate nanoregions may act as nucleation sites for the calcium phosphate-rich layer. Later on, this group investigated the nanostructure of SiO_2_–P_2_O_5_–CaO S-GG [23]. The study showed how the incorporation of calcium into the glass promoted the appearance of P–O Nonbonding Oxygen (NBO) groups and that the glass nanostructure was induced to form Ca-rich orthophosphate units.

In 2013, the structures of binary CaO–SiO_2_ and ternary CaO–P_2_O_5_–SiO_2_ S-GG by MD simulations, using the melt-quenched and sol-gel protocols, were investigated [24]. The combined effects of the silicate network connectivity and the tendency to form or not form non-homogeneous domains were correlated. The composition with an optimal Ca/P ratio was synthesized and the in vitro tests confirmed the prediction by MD. Furthermore, in a recent paper, the advantages of using reactive MD models of S-GG were discussed [25].

The influence of the nanostructure in the bioactive response was also investigated for other families of silica-based biomaterials, like the CaO–SiO_2_–PDMS hybrid sonoaerogels [26]. The aerogels showed analogous microstructural features to BG exhibiting amorphous Ca-free areas where the Si–O–Si distances were 0.23 nm. Besides, Ca-containing crystalline nanodomains, of pseudowollastonite, CaSiO_3_, were detected in the hybrid containing 20% CaO and 20% PDMS, the only one of the investigated series that exhibited a bioactive response in SBF.

### 3.4. Nanostructural Characterization of Bioactive Glasses by Nuclear Magnetic Resonance

Solid state NMR is also playing an important role in the characterization of the nanostructure of BGs and the relationship with their behavior in SBF. Besides the structural characteristics of BGs, which will be explained in this section, NMR was used to identify the material formed on the glasses when immersed in SBF, which will be the subject of the following section. Next, outstanding advances on the nanostructure of different BGs where NMR spectroscopy played a fundamental role are presented.

**Figure 6:**
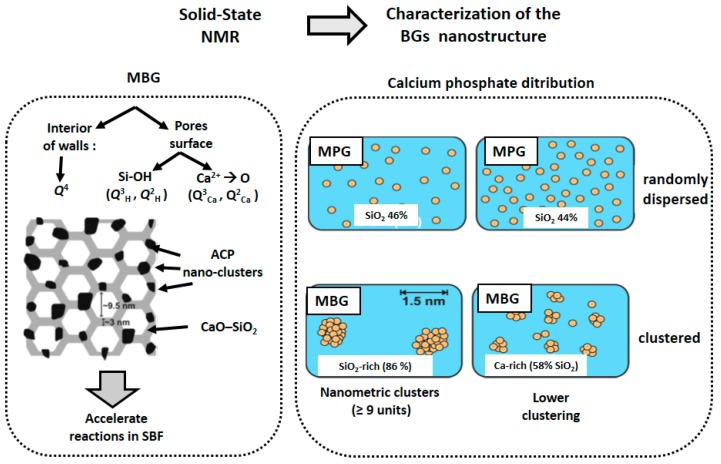
Solid state NMR allowed the determination of nanostructural features of BGs influencing the behavior in SBF. **Left**: The proposed model for MBGs contains ACP nanoclusters able to accelerate the reactivity in SBF. **Right**: NMR was also able to establish differences between MPGs and MBGs. In MPGs, phosphate ions are randomly dispersed, whereas in MBGs, nanometric clusters are formed whose size increases when the SiO_2_ in the glass increases.

In 2008, Leonova et al. [27] investigated for the first time the local structures of CaO–SiO_2_–P_2_O_5_ MBGs with variable composition by ^1^H, ^29^Si and ^31^P NMR. A biphasic structural model was established (see Figure 6, left). Such a model was valid with minimal differences for the MBG composition richer in Ca (35.2 atom-%) and for the two compositions with lower contents in this element (19.1 and 9.5 atom-%). The authors highlighted that the pore wall leads to a high accessibility of Ca and P to body fluids and related this model to the experimentally demonstrated high bioactivity in SBF for these materials.

Later on, in 2012, Gunawidjaja et al. [28] established a model showing the presence of phosphate nuclei on the MBG structure. They showed the advantages of using ^29^Si and ^1^H NMR spectroscopy for probing the local structures of bulk and surface portions of MBGs. ^29^Si NMR spectra were obtained in conditions that allowed them to obtain quantitative information about the silicate speciation in the pore wall and at the MBG surface. The MBG surface was monitored after being in SBF for periods of time of up to 7 days. A depletion of Ca^2+^ ions at the MBG surface and a minor condensation of the silicate surface network over 7 days in SBF were observed. In 2013, this group probed the phosphate–ion distribution in some bioactive MPGs and MBGs by ^31^P NMR [29]. The occurrence of transitions between random and clustered scenarios was evidenced (Figure 6, right). Phosphate species were randomly dispersed in Ca-rich MPGs (SiO_2_ from 44 to 55 mol-%), whereas Ca-poor MBG structures (86 mol-% SiO_2_) exhibited nanometric ACP clusters, comprising at least nine orthophosphate groups. In addition, a Ca-rich MBG (58 mol-% SiO_2_) revealed a less pronounced phosphate clustering.

On the other hand, Fayon et al. [30] determined in 2013 the distribution and nature of nanometric-sized phosphate clusters in a bioactive MPG by ^31^P NMR. It must be taken into account that dissolution of phosphates in MPGs raises the question of chemical homogeneity and the possible formation of clusters. Furthermore, the presence of phosphate clusters of 5 and 6 [PO_4_] tetrahedral units embedded in the disordered polymeric silicate network was detected. In 2014, Svensson et al. [31] used simultaneously ^31^P NMR and MD simulations to determine the phosphate distribution in eleven bioactive and non-bioactive phosphosilicate MPGs. They present an assessment of the distribution of phosphate groups across the structures, with the P content and silicate network connectivity varying independently.

## 4. Characterization of HCA Formed In Vitro on Bioactive Glasses Mainly Using NMR

NMR can also be used to analyze the HCA formed in SBF, allowing the identification of the formation steps of the calcium phosphate layer on BGs. Thus, in 2012, Gunawidjaja et al. [32] compared the biomimetic growth of HA from a MBG in SBF and buffered water by solid state NMR, powder XRD, SEM, and EDX. The effect of using two MBG concentrations was examined. The relative amounts of ACP and HA were quantified by XRD and ^31^P. For high MBG loadings, the HA formation reduced in SBF compared to buffered water. These features stem from a high [Ca^2+^]/[PO_3_^4−^] ratio resulting in the medium, which retards the HA crystallization by inducing a rapid ACP precipitation.

**Figure 7:**
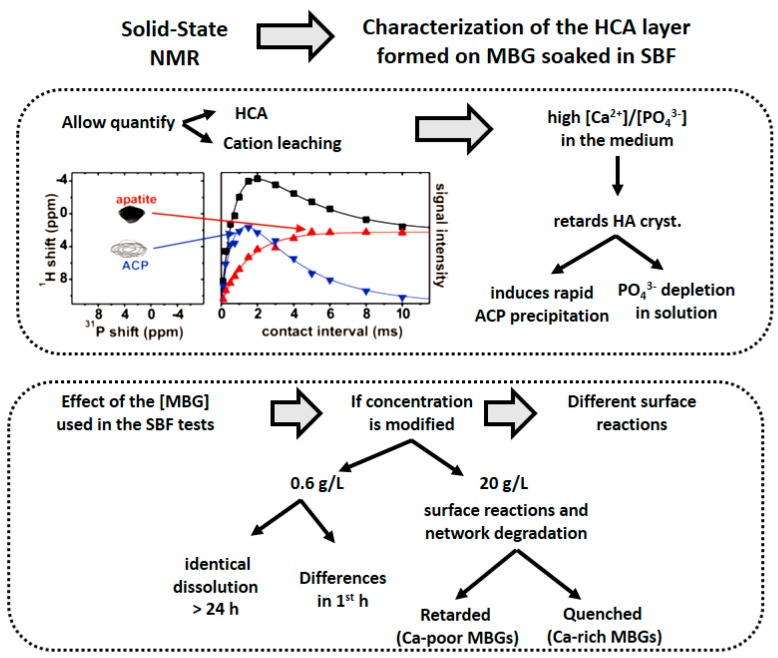
NMR can be used to characterize the HCA coating the bioactive MBG after being soaked in SBF. Top: The relative amount of ACP and HCA in the newly formed layer was quantified. NMR demonstrated that high Ca^2+^/PO_4_^3−^ ratios in the surrounding liquid medium retarded the HCA formation on MBG. Bottom: NMR demonstrated the effect of the MBG concentration used in the SBF tests.

In 2015, the composition-dependent in vitro formation of apatite at MBG surfaces was quantified by ^31^P NMR and XRD [33]. That way, the first quantitative assessment of the HCA growth in SBF from a BG was reported (Figure 7, top). The authors determined a profile of the relative ACP/HCA fractions layer formed at each MBG and assay time. The HCA in each BG after the SBF treatment and the composition of Ca-depleted MBG phase were determined from XRD in conjunction with measured concentrations of Ca, Si, and P in solution. The HCA formation observed increased concurrently with the Ca and P contents of the MBG. The results of this study highlight the importance of adapting the concentration of the biomaterial to its composition to avoid perturbing the HCA crystallization, altering the outcome of the SBF assays.

In 2016, the surface reactions of MBGs monitored by ^29^Si NMR and the effects of particles of glass concentration in SBF were reported (Figure 7, bottom) [34]. MBG revealed different surface alterations between low (0.6 g/L) and high (20 g/L) MBG concentrations in SBF. For the low MBG dose, expected to be more relevant for in vivo conditions, all MBGs followed a similar dissolution mechanism beyond 24 h. In contrast, for high MBG concentrations, the surface reactions and their associated silicate network degradation were retarded for Ca-poor MBGs, whereas the reactions were completely quenched for Ca-rich compositions. These findings simplify future MBG design by identifying the compositional and textural factors relevant for a rapid in vitro formation of HCA.

In 2017, Mathew et al. monitored the proton environments in calcium phosphates formed in vitro from MBGs by ^1^H NMR [35]. The evolution of the proton speciation was elucidated at the MBG surface and within each ACP/HCA constituent of the layer formed after immersed in SBF. The initially prevailing ACP phase comprises H_2_O HPO_4_^2−^/PO_4_^3−^ groups in a non-apatite structure. When the soaking time increased, a transformation of ACP to HCA was evidenced. They demonstrated that ^1^H ^31^P cross-polarization NMR was more sensitive than powder XRD or ^31^P NMR for detecting the onset of HCA formation, mainly for P-containing MBGs.

## 5. Future Perspectives and Conclusions

Determining the structure of glasses on the nanoscale is essential to understand why some glasses are coated with an HCA layer when soaked in fluids that simulate human plasma. Furthermore, it is necessary to understand the different bioactivity mechanisms observed, as a function of the presence or absence of phosphorus in the glasses’ composition.

After the first paper describing the nanostructure of two BGs [15], many research groups investigated the glasses’ characterization on the atomic scale by experimental techniques including HRTEM, MD, and NMR. The final aim was to establish relationships between the glasses’ nanostructure and the bioactive behavior. These studies, extended to the three families of bioactive glasses, i.e., MPGs, S-GGs, and MBGs, allowed the elucidation of unknown aspects of the apatite layer formation under in vitro conditions. In P-containing BGs, the nanostructural characterization revealed that Ca^2+^ ions are out of the glass network and bonded to phosphorus, forming silicon-doped calcium phosphate nanocrystals. This fact explains the different mechanisms of formation of the apatite layer depending on the presence or absence of P in the glass.

**Figure 8:**
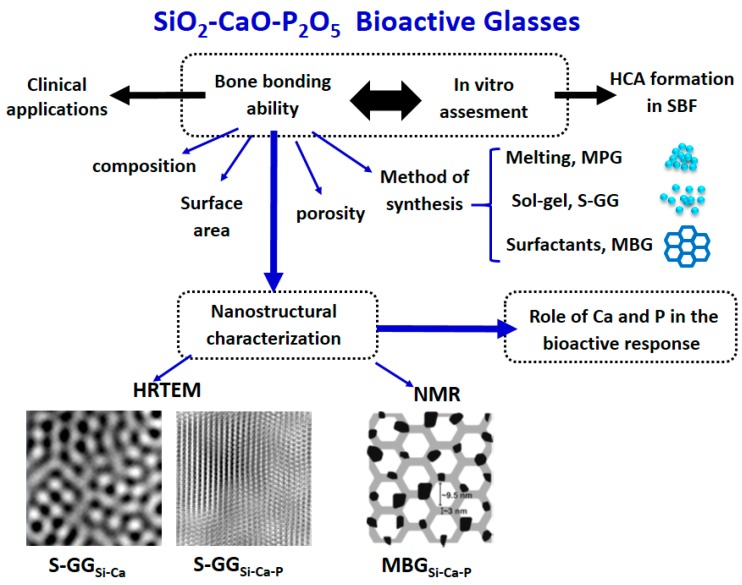
The nanostrucural characterization of glasses allows the explanation of the different mechanisms of formation of the HCA layer after being soaked in SBF. The relationship of composition and textural properties with the in vitro bioactive response was profusely investigated, but the nanostructural features of the BGs also exert a critical role in the glasses’ reactivity.

Figure 8 summarizes the properties of glasses influencing the in vitro behavior in SBF. Besides composition, method of synthesis, and textural properties (for S-GGs and MBGs), the nanostructural characterization plays an important role in the design of new BGs for tissue engineering because it allows the prediction of its behavior in the human body when implanted.
